# Autopsy Revealing the Absence of Gall Bladder

**Published:** 1850-10

**Authors:** 


*Autopsy revealing the Absence of Gall Bladder.* By D. B. TRIMBLE, M. D., of Burlington, N. J.—Under the impression that whatever will tend to enlighten us, in relation to the often obscure symptoms of disease, is worthy a place in the records of medical science, however humble may be the effort, I send for insertion in the Reporter, an account of a post-mortem examination of some interest.

The subject of examination was a woman, aged fifty-five years, whom I was called upon to attend, on the sixth of January.

I found her complaining of pain in the epigastric region, nausea, occasionally vomiting, slightly furred tongue, pyrosis, anorexia, and constipation. All these symptoms had been progressing for several months, though she was still able to attend to her household duties. Firm pressure on the epigastric region gave her pain, but not acute. Her pulse was frequent, though not full, or tense. As her disease progressed, she twice, at considerable intervals, and for some hours, complained of pain in the shoulder; her appetite became more and more depressed; emaciation increased rapidly, ascites supervened, and death released her on the 24th of June.

From the symptoms, I considered it a case of chronic gastro-enteritis, approaching the stage of ulceration, and the treatment was in accordance with these views. The autopsical examination, however, showed that my diagnosis was partially wrong, but at the same time proved that no medical efforts would have relieved her.

On the evening of the 24th, accompanied by my friend Dr. Gauntt, I proceeded to the investigation, and on opening the abdomen, found it filled with serous fluid, of a reddish color, in quantity about one gallon. The colon, and the small intestines, were lying exposed, and the only appearance of an omentum was a strip about an inch in width, attached to the stomach. We next examined the intestines, commencing at the rectum, which was filled with ash-colored fæces, and inflamed. The cæcum, though less inflamed than the other intestines, had bright red patches throughout its coats; the colon, jejunum, and ileum, showed strong evidences of inflammation, being of a dark mahogany color, and the coats of the small intestines much thickened. The mesenteric glands were discolored, but not enlarged. The duodenum was much contracted in size, and, about that portion of it where the pancreatic duct, and the ductus choledochus penetrates it, was adherent to the liver and pancreas. The *stomach* presented no signs of disease. The liver was diminished in size, the stomach being uncovered by the left lobe; the right lobe was also smaller, and the portion attached to the pancreas together with that viscus, were of a cartilaginous character. No *gall-bladder*, or any vestige of one, could be found, after the most careful investigation. Upon opening the cartilaginous portion of the liver, a gall-stone was found imbedded in the ductus choledochus, just at its entrance into the duodenum. It measured in its long circumference two and three-quarter inches; in the small circumference two inches, and weighed one hundred and nine grains. It is of a dull red color with patches of a white crystaline coating. The uterus was healthy in appearance, though filled with a semi-organized substance. Between the insertions of the round ligament, and the fallopian tube of each side, was a tumor, on the left side about the size of a large pea, and of an osseous consistence; on the right as large as a hazel-nut, and cartilaginous. They were so situated, that the slightest pressure on them would close the orifices of the fallopian tubes. She had been married twenty-five years, but had not conceived. Could these tumors, by their mechanical effect, be the cause? The ovaria were healthy.

She had suffered for some years with the usual symptoms of indigestion; but had not, at any time, very acute pain in the *right* hyyochondriac, or epigastric regions; at least none that could cause a suspicion of the passage of a calculus of the ordinary size.

From the absence of the gall-bladder, no cystic bile could have been secreted; and from the situation of the stone, no bile could pass into the duodenum, thus seriously deranging digestion and nutrition, producing the inflammatory condition of the intestines, resulting in death, and the *cause* of which, no art could remove.

The questions arise, what became of the omentum and gall-bladder?—or was this one of those abnormal formations mentioned by authors, where those organs are absent? If there was originally no gall-bladder, how was the calculus formed? or did its size produce ulceration and rupture of the bladder, and its subsequent absorption? These are interesting questions, but difficult of solution.

Upon mentioning the foregoing case to a medical friend from Philadelphia, he informed me that he recently had a case of calculi in the gall-bladder and ducts, resulting in death in fifteen hours, in which there was no pain in the *right* hypochondrium; but intense pain in the *left*, extending to the epigastrium. Two of the most eminent of the physicians of that city, saw her with him, and were not led by her *symptoms* to suspect her disease, though one of them suggested that such might be the cause. The post-mortem examination disclosed the gall-bladder filled with small calculi, one of which, not larger than a small pea, had closed the passage of the cystic duct. These cases show the obscurity of the diagnosis in biliary calculi, and should lead us to suspect their presence when efficient means are unavailably used to relieve affections of the stomach and bowels, when timely resorted to.—*New Jersey Med. Reporter.*

